# Supplementary material for: Parp1 hyperactivity couples DNA breaks to aberrant neuronal calcium signalling and lethal seizures
Source: EMBO Rep. 2021 May 1;22(5):e51851. doi: 10.15252/embr.202051851 (PMC8097344; doi:10.15252/embr.202051851)
Supplement: Supplementary file 1 — Expanded View Figures PDF [file EMBR-22-e51851-s002.pdf]

Expanded View Figures

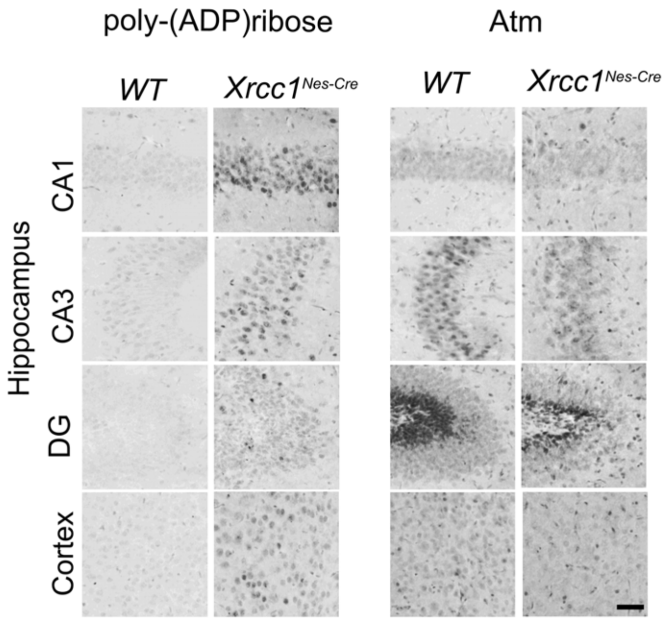

Figure EV1. Levels of poly(ADP-ribose) and Atm in Wild-type and *Xrcc1*$^{Nes\text{-}cre}$ brain.

Sagittal sections obtained from mice (p15) of the indicated genotypes were immunostained using anti-poly(ADP-ribose) antibody (Trevigen; 4336) or for Atm protein using the antibody EPR17059 (Abcam; ab199726). Representative images of the hippocampal regions CA1, CA3 and dentate gyrus (DG), and in the cerebral cortex, are shown as indicated. Scale bar 50 μm.

© 2021 The Authors
